# Reweaving the Tapestry: a Supertree of Birds

**DOI:** 10.1371/currents.tol.c1af68dda7c999ed9f1e4b2d2df7a08e

**Published:** 2014-06-09

**Authors:** Katie E Davis, Roderic D. M. Page

**Affiliations:** Department of Biology & Biochemistry, University of Bath, Bath, UK; Institute of Biodiversity, Animal Health and Comparative Medicine College of Medical, Vetinary and Life Sciences University of Glasgow, Glasgow, UK

## Abstract

Our knowledge of the avian tree of life remains uncertain, particularly at deeper levels due to the rapid diversification early in their evolutionary history. They are the most abundant land vertebrate on the planet and have been of great historical interest to systematists. Birds are also economically and ecologically important and as a result are intensively studied, yet despite their importance and interest to humans around 13% of taxa currently on the endangered species list perhaps as a result of human activity. Despite all this no comprehensive phylogeny that includes both extinct and extant species currently exists. Here we present a species-level supertree, constructed using the Matrix Representation with Parsimony method, of Aves containing approximately two thirds of all species from nearly 1000 source phylogenies with a broad taxonomic coverage. The source data for the tree were collected and processed according to a strict protocol to ensure robust and accurate data handling. The resulting tree topology is largely consistent with molecular hypotheses of avian phylogeny. We identify areas that are in broad agreement with current views on avian systematics and also those that require further work. We also highlight the need for leaf-based support measures to enable the identification of rogue taxa in supertrees. This is a first attempt at a supertree of both extinct and extant birds, it is not intended to be utilised in an overhaul of avian systematics or as a basis for taxonomic re-classification but provides a strong basis on which to base further studies on macroevolution, conservation, biodiversity, comparative biology and character evolution, in particular the inclusion of fossils will allow the study of bird evolution and diversification throughout deep time.

## Introduction

The class Aves contains an estimated 10,000 extant species^1^ occupying almost every geographical location, from ocean to desert. They originated within theropod dinosaurs during the Jurassic period^2^, with the earliest recognised stem group bird being the iconic *Archaeopteryx lithographica*, of which a number of 150 million year old fossils have been discovered in the famous Solnhofen lagerstätte of Germany. Regular new discoveries, particularly from the vertebrate rich Cretaceous deposits of China, continue to improve our understanding of the earliest birds^3^^,^ 4 ^,^ 5. Modern birds experienced a rapid radiation early in their evolutionary history, though the timing of this is contentious^6^, resulting in the remarkable diversity that we see today. This rapid radiation of deeper branches is, however, the main confounding factor in our attempts to find the “true” avian tree of life.

Birds are of great interest in a range of fields such as comparative biology, conservation and macroevolutionary studies. They are an economically important group, providing food for humans, as well as fertilizer, and some species are kept as pets. Yet human activity may be partly to blame for the 1,313 species currently on the IUCN Red List of threatened species^7^; there is a real risk of extinction to many bird species and much effort is being directed towards issues in conservation. Phylogenies are an important tool in conservation, as highlighted by Nee and May^8^, and allow testing of hypothetical extinction models to assess the loss of “phylogenetic diversity". Large, well-resolved phylogenies are also vital when attempting to answering important macroevolutionary and macroecological questions yet surprisingly few attempts have been made to reconstruct the phylogeny of all birds and no comprehensive tree including fossil taxa has yet been published though it is well-known that the exclusion/inclusion of fossil taxa can have implications on the resulting phylogenetic tree. Many previous comparative studies have been based on Sibley and Ahlquist’s “tapestry”^9^, constructed using the much-criticised technique of DNA-hybridisation. Although a massive achievement at the time, this phylogeny contained just 1083 taxa, around a quarter of all birds, with most taxa at genus-level. A number of comparative studies using birds have been based on the tapestry of Sibley and Ahlquist; these include the tempo and mode of bird evolution^10^, the effect of generation time on rates of avian molecular evolution^11^, the evolution of avian mating systems and the association between mating systems and pair-bond length^12^. The dependence of these comparative analyses on the tapestry is troubling as there are concerns about the validity of the method used^13^^,^ 14 ^,^ 15. Although there have been more recent attempts at an inclusive bird phylogeny based on large molecular data sets these are still largely incomplete^16^. A recent large phylogeny of birds^17^ contains 9993 taxa but the use of the results of previous studies as a backbone is potentially problematic. In addition, approximately one third of the taxa were added manually *post priori*. These recent attempts were based on molecular loci and therefore, by definition, excluded extinct taxa. The inclusion of fossils is vital for macroevolutionary studies and investigations into the origins of modern birds.

There are two approaches used for creating large phylogenies. One is the supermatrix or “total evidence” method^18^^,^ 19 ^,^ 20. Here, all characters and taxa make up a single large matrix. A major drawback of this approach is that some types of data cannot be combined (e.g. immunological distance data and DNA-hybridisation data) and that combination of these data types introduces subjective decisions and is vastly time consuming. There is also the potential for a large amount of missing data when combining information in this way^21^. Bird systematists have employed hard and soft body morphology, behaviour, allozymes, nucleotide sequences, and DNA-hybridisation to elucidate avian phylogeny. Consequently, a supermatrix approach would *a priori* eliminate many of these data sources. Supertree methods offer a practicable approach to synthesising large numbers of smaller overlapping phylogenies. These “source trees” are built with primary data (e.g., character sets obtained from morphological features or from gene sequencing) or and can be constructed using any method and contain any number of taxa. Many supertree methods also allow conflict between source trees. Therefore supertrees give the widest possible view of phylogeny, both in terms of taxonomic coverage and the types of data incorporated. There are some well-documented issues in using supertree methods to construct large phylogenies, such as data quality and the reliance upon secondary rather than primary information^22^^,^ 23. We attempt to minimise these issues where possible by the use of a strict and robust data processing protocol^22^^,^ 24. Whilst this will not eliminate all possible issues, it allows the construction of an inclusive and large phylogeny. In the future combining supertree and supermatrix methods to complement each other is a potential solution to resolve some of the pitfalls of each method^25^.

Supertrees have now been produced for many groups of taxa including dinosaurs^26^, tetrapods^27^^,^ 28, grasses^29^, mammals^30^ and crocodiles^31^. Supertrees have also been produced for avian subsets such as the tube-nose seabirds^32^, shorebirds^33^, oscine songbirds^34^, the fowls^35^, and a 980 taxon supertree across all extant orders^36^ but no comprehensive supertree has yet been constructed for all of Aves. Our aim is to combine data from all sources, including both fossil and extant taxa, to create an inclusive phylogeny of birds that will help elucidate their origins and aid conservationists in concentrating their efforts in preserving so-called “biodiversity hotspots".

## Methods

**Source tree collection**

Potential source trees were identified initially from online resources. The Web of Knowledge Science Citation Index (http://wok.mimas.ac.uk) was searched using the search terms: phylog*, taxonom*, systematic* and clad* in conjunction with all scientific and common names of birds from order to family level. These searches were carried out from the year 1976 up to 2009. This is a significant update - an additional two year's worth of data (118 published papers) - compared to the tree of Davis^24^. See conclusions for further comments on the scope, and limitations, of our search. Following the initial search all papers potentially containing phylogenetic trees were examined. In addition, the reference lists of these papers were checked to obtain any further potential source trees. All source trees, along with associated meta-data, were recorded in their original form exactly as they appeared in the source references. Meta-data includes information about source trees such as bibliographic details, characters used (molecular, morphological, behavioural etc.), methods used for tree building, and the taxa included in the analysis. These data were stored in XML file format while the trees were recorded using TreeView^37^. At this stage no corrections were made for synonyms or any other apparent errors or inconsistencies in the source trees.

Data quality is a big challenge in supertree construction^22^^,^ 23 therefore a strict protocol for data processing was implemented based on that first described by Bininda-Emonds *et al*.^22^ . This protocol was followed with some modifications^24^ and implemented using the Supertree Tool Kit (STK) software^38^. Source trees needed to meet several criteria for inclusion in the analysis: 1) it should be explicit that the author’s intention was to construct a phylogeny, 2) the characters and taxa used in the analysis must be clearly identifiable and 3) the tree should be based on an analysis of a novel, independent dataset. We defined non-independence as two or more studies that use the same character data and have identical taxa or two or more studies that use the same character data and where one taxa set is a subset of the other. In the case where one was a subset of the other the less comprehensive tree was removed from the dataset. Where this was not possible trees were combined to create a single tree for inclusion in the supertree analysis. Identification of potentially redundant data was automated through use of the STK software. Despite our first criterion we included a taxonomic backbone or “seed tree” as a source tree as studies have shown that the inclusion of a large taxonomically complete greatly improves the performance of supertree methods^39^. This approach is far more conservative than placing constraints on the dataset; the poorly resolved taxonomy tree acts only to improve overlap and will not over-ride stronger signals within the dataset. We created a very conservative tree, only including taxa that could be unambiguously assigned at order level, compiled using Howard and Moore Catalogue of Birds of the World^40^. Orders that are in a state of flux were either excluded entirely, (e.g., the “Bucconiformes”) or in other cases the core members of the order were included but taxa whose membership of that order is uncertain were excluded (e.g., the Pelecaniformes). Fossil taxa were added to the backbone tree using the Paleobiology Database (paleodb.org) as a guide.

**Nomenclature and taxonomic consistency**

OTUs (operational taxonomic units) were standardised to avoid the inclusion of higher taxa and vernacular names that would artificially inflate the number of taxa in the analysis and synonyms and misspellings that could lead to inconsistencies. Names were standardised according to Howard and Moore^40^, chosen for its conservative approach. Paraphyletic taxa were dealt with using the STK^38^, which calculates all possible positions of paraphyletic taxa in a source tree. Once all the possible, non-identical, permutations have been calculated, a mini-supertree can be created from them. Higher taxa and vernacular names were removed from source trees by substituting the constituent taxa in a polytomy. Where possible the actual species that the authors intended to represent were used. Where this was not indicated in the source reference all taxa that make up the higher taxa or vernacular name were used but only those that were already present in the dataset to avoid artificial inflation of the number of taxa. Definitions for higher taxa were also according to Howard and Moore. Some substitutions were necessarily very large, for example, a number of source trees contained the taxon “Neornithes” which requires the substitution of virtually every taxon contained within the supertree. The STK contains a tool that enables these to be substituted automatically using a text file containing a user-defined list of substitutions. This substitution stage should not introduce taxa that are not already contained within the dataset, the STK deals with this by checking the presence/absence of substituted taxa by checking the substitution file that is to be used against the source data and indicates any potential problem taxa. The final step for taxonomic/nomenclatural standardisation is the replacement of generic taxa to specific, again in the form of a polytomy containing all taxa of a given genus with the caveat that they are already present in the dataset. Once nomenclature had been standardised it was possible to check that the source trees have sufficient taxonomic overlap, we required each source tree to have least two taxa in common with at least one other source tree^21^. After all data processing had been completed checks were carried out to ensure that no errors had been introduced during data processing. Again this was implemented using the STK which checks the tree files against the meta-data held about the source trees. This guards against both software and human errors. After these final checks the dataset contained 6326 taxa from 1036 source trees. See additional file 1^41^ for a list of papers containing the source trees and additional file 2^41^ for a Nexus file containing all source trees.

**Supertree construction**

The most commonly used supertree method is Matrix Representation with Parsimony (MRP)^42^. Although more supertree methods have become available over the last few years, many of with them software implementation (e.g., SuperFine^43^, Matrix Representation with Compatibility^44^, Minimum Flip^45^, Modified MinCut^46^), they tend to be slow and unable to deal with large datasets within a reasonable time frame. We chose to use MRP for this analysis as it is still the only supertree construction method able to deal with a dataset of this size. In matrix-based supertree methods all taxa subtended by a given node in a source tree are scored as “1”, taxa not subtended from that node are scored as “0”, taxa not present in that source tree are scored as “?”. Trees are rooted with a hypothetical, all-zero outgroup^47^. We used standard Baum and Ragan MRP coding^42^ and the matrix creation was automated using the STK software^38^. See additional file 3^41^ for the matrix in TNT format.

The matrix was analysed with TNT^48^ using the “xmult level=10” option, an aggressive search strategy devised to find the shortest trees in as little time as possible. The analysis was run on the Imperial College supercomputer CX1. We ran the analysis on 100 cores for 144 hours, which is equivalent to over two years of computational time on a single core. Each core ran an independent analysis, using a different random starting point for the heuristic search. In this way as much of the phylo-space as possible can be covered in as short a time as possible.

Support values were not calculated for the supertree. An attempt was made to calculate QS values^49^ for the tree, however CX1 ran out of memory after five days and was unable to complete the analysis. The calculation of V values^50^ faced similar computational limitations. Traditional support measures such as bootstrap and jack-knifing are of debatable relevance to supertrees^51^ and would face the same computational limitations.

## Results

The analysis found nine MPTs of length 28834 (additional file 4^41^ ). Some areas of the tree were poorly resolved with some odd taxon placement, on closer inspection many of these taxa were observed to be those that are poorly constrained within the source trees or poorly represented within the dataset (see discussion) therefore we calculated an agreement subtree. We were unable to calculate a Maximum Agreement Subtree in PAUP* 4.0b10^52^ due to memory limitations so we used the Approximate Agreement Subtree function in TNT^48^. This function uses a heuristic that accurately obtains an agreement subtree but does not guarantee to find the one with the greatest number of taxa (i.e. the MAST). The agreement subtree contained 5380 taxa and the resolution was greatly improved. Figure 1 shows the whole supertree with higher taxa indicated. This figure gives an indication of the size of the tree and the relative sizes of clades. For a simplified order-level tree see Figure 2. For an electronic version in which the whole tree can be viewed in detail see additional file 5^41^

**Agreement subtree calculated for nine MPTs of length nine MPTs of length 28834.**
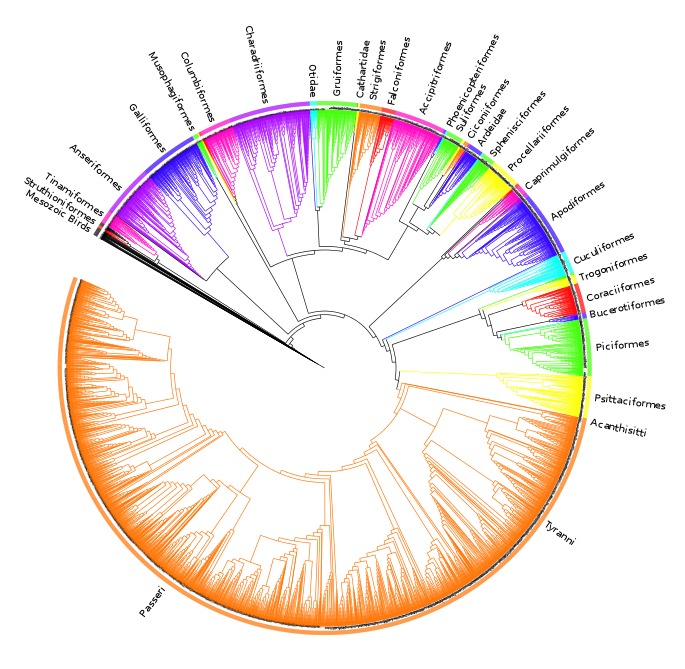

**Simplified summary supertree showing order-level relationships:**
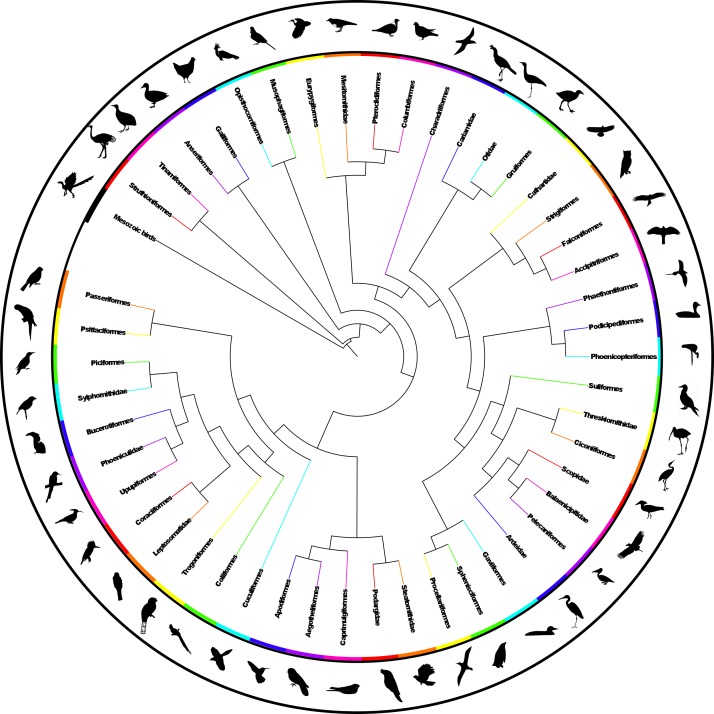
Silhouettes are from http://phylopic.org.

## Discussion

**Taxonomic coverage, data types and resolution**

The resulting supertree contains approximately two thirds of all known birds synthesised from source data from the years 1976 to 2008. The number of source trees from each year shows that the majority of data are derived from 2000 onwards (Figure 3). The vast majority of the source data comes from molecular sources (Figure 4) with cytochrome b being the single highest contributor to the data set with 38.9% (403 trees) of source trees built from cytochrome b sequences. See additional file 6^41^ for further information on the composition of the data set. Published molecular studies more than doubled in the period from 2000 to 2009, quickly becoming the largest source of available data. The overall topology of the supertree is more consistent with molecular hypotheses, possibly due to the strong bias towards molecular analyses in the source data. Figure 5 shows the overall distribution of taxa sampled in source trees in the form of a data availability matrix. The density of data sampling is excellent with a large densely sampled area and very few trees and taxa with poor sampling. Resolution of the tree is very high (99.85%).

**Distribution of source trees by year of publication.**
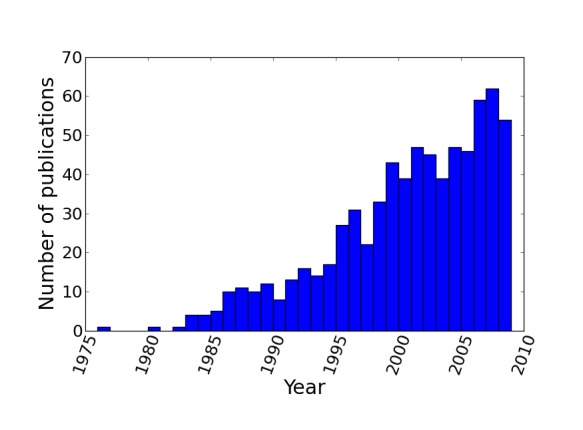
The number of avian phylogenies published, and included in the supertree analysis, is heavily skewed towards recent years with relatively few trees from pre-1995.

**Distribution of source trees by year of publication.**
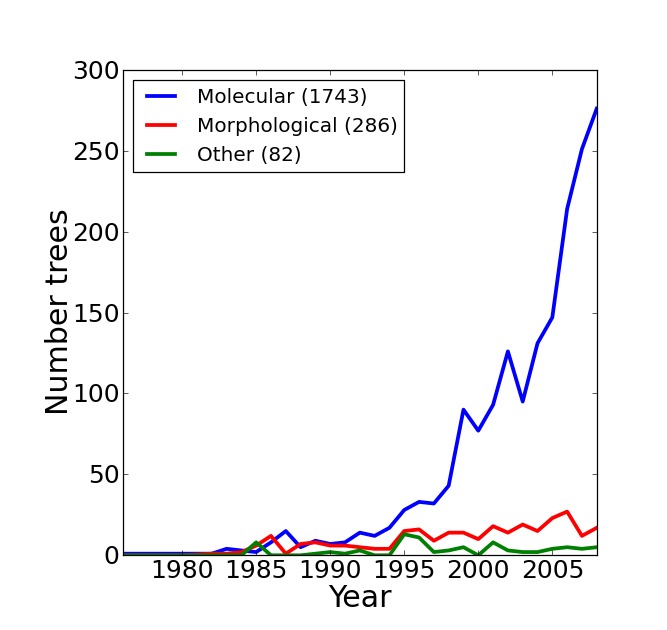
The number of avian phylogenies published, and included in the supertree analysis, is heavily skewed towards recent years with relatively few trees from pre-1995.

**Data availability matrix for the supertree source data.**
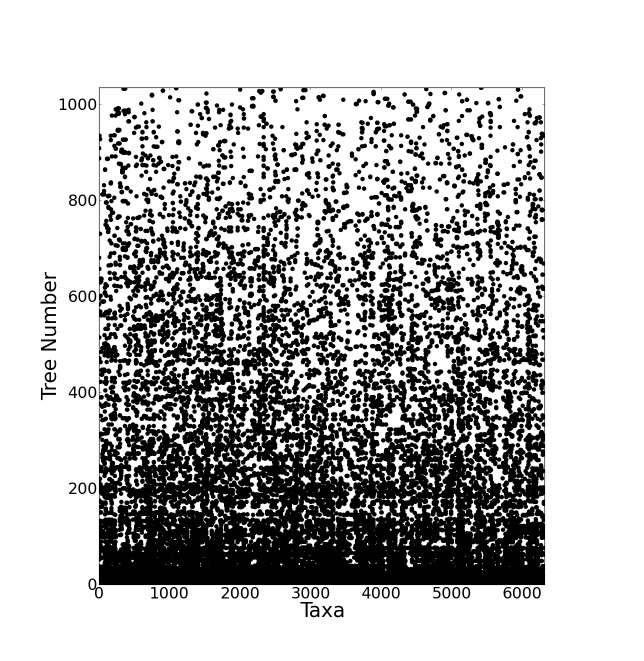
Source trees are sorted vertically and taxa are sorted horizontally both by frequency. Each dot represents the presence of a taxon in a given source tree. The most frequently occurring taxon, *Gallus gallus*, is at the bottom. The bottom left hand corner shows the most densely sampled area where many taxa are found in many source trees.

**MRP performance and novel clades**

The MRP method cannot provide new information on relationships that is not already present in the source trees but it is a convenient and fast method for summarising the current state of phylogenetic knowledge. Although some spurious relationships may be recovered, eg., novel clades as discussed below, the majority of the relationships found in the supertree are well-supported by the source trees (see main discussion for details).

A small number (approximately 3%) of taxa are placed in novel clades; i.e. clades that are not supported by the source data. These novel clades tend to occur at the bases of large clades near the tips of the trees and therefore only affect the lower level relationships. The vast majority are found within the order Passeriformes, an order that has historically posed the biggest challenge to avian systematists^34^. An examination of these taxa and the corresponding source trees showed that these taxa, without exception, are characterised by one or more of the following:

1. Presence in only a small number of source trees.
2. Variable position within source trees.
3. Commonly placed in source trees as part of a polytomy.
4. Often/only present as an outgroup.

See additional file 7^41^ for a list of problematic taxa and their occurrence within the source trees. Post-Mesozoic fossil taxa are particularly poorly represented by the source data and as a result are commonly found in novel clades. *Palaeopsittacus* and *Psittacopes* for example are each only represented by one source tree^53^^,^ 54, therefore the algorithm is unable to accurately place them. Another observation made is that MRP has a tendency to place fossil taxa in highly derived positions, e.g., the Cretaceous anseriform fossil *Vegavis* is placed within a clade of extant ducks despite there being no source trees to support this relationship. These poorly constrained taxa are a big problem for supertree analyses and these “novel clades” are a well-known, but problematic, property of Matrix Representation with Parsimony^49^. Algorithms and software are becoming available to help reduce this problem by identifying potential rogue taxa either prior to running an analysis or *post priori* 55 but, as is the case with support values, they cannot cope with the size of the present dataset and we find that the analysis is again necessarily limited by computational constraints. The size of the data set also makes manual identification of these taxa, as we have done here, extremely time-consuming and cumbersome.

Another problem is that many of the algorithms available only identify taxa that may be placed incorrectly as a result of many possible, equally parsimonious positions in the tree (e.g., Safe Taxonomic Reduction^56^) but this does not appear to be the sole problem with MRP and what is really needed is a leaf-based measure of support that would readily identify potential rogue taxa that occupy positions in the tree for which there is no evidence in the underlying data. In the meantime we suggest caution is used, particularly if fossil taxa are to be used for obtaining clade divergence dates.

**Tree topology**

The tree is well-resolved and stable at both order and family level with the majority of families and orders resolved as monophyletic; see discussion below for exceptions.

Deep divergences

The extinct Mesozoic birds are placed at the base of the tree with* Archaeopteryx lithographica* occupying the most basal position. Within these the Enantiornithes (“opposite birds”) form a distinct monophyletic clade. The Enantiornithes represent a separate radiation to the Ornithurae (the direct ancestors of modern birds) that subsequently became extinct at the Cretaceous-Tertiary boundary^57^^,^ 58 ^,^ 59 ^,^ 60. The earliest divergences of birds are amongst the least controversial^61^ and here the supertree supports the split of the crown group modern birds (Neornithes) into Palaeognathae + Neognathae with a further spilt of Neognathae into the Galloanserae landfowl/waterfowl clade + all other modern birds (Neoaves)^62^^,^ 63 as opposed to Sibley and Ahlquist’s^9^ non-monophyletic Neognathae in which the Galloanserae are sister group to the Palaeognathae.

Palaeognathae

The extinct Tertiary palaeognaths *Lithornis* and *Palaeotis* are basal to the extant palaeognath taxa. The supertree supports the hypothesis of Tinamidae + all other palaeognaths^64^. The extinct Madagascan elephant bird *Aepyornis* appears within the Struthioniformes at the base of the Struthionidae + Rheidae while the Dinornithidae of New Zealand appear at the base of the Struthioniformes clade. The New Zealand ratites, Apterygidae and Dinornithidae, do not form a monophyletic group. This has been suggested by Houde^14^ and Cooper *et al*.^65^ to have implications for vicariance biogeography providing evidence for a second colonisation of New Zealand by kiwis.

Galloanserae

The Galliformes + Anseriformes clade is well-supported by molecular works^66^. Within the Galliformes, the supertree supports the more recent view of Megapodidae as sister to the Cracidae + remaining Galliformes, which all constitute monophyletic families rather than the more traditional placement of a Megapodidae + Cracidae clade as sister to the rest of the Galliformes^9^. The Anseriformes are split into the three well-defined traditional, monophyletic families: Anhimidae, Anseranatidae and Anatidae.

“Waterbird” assemblage

The supertree recovers the “waterbird” clade containing the “Pelecaniformes”, “Ciconiiformes”, Procellariiformes, Sphenisciformes and Gaviiformes as found in the large molecular analyses of Ericson *et al*.^67^ and Hackett *et al.* 16. Morphological evidence also supports this clade^68^. The Livezey and Zusi phylogeny^68^ also places the orders Phoenicopteriformes, Podicipediformes and Phaethontiformes within this assemblage, as can be seen in the supertree.

Within this assemblage the traditional “Pelecaniformes” are split into two groups, one comprising the Pelecanidae, the other the Fregatidae + Sulidae + Anhingidae + Phalacrocoracidae. In addition the Pelecanidae are grouped with the “ciconiiform” families Ardeidae, Balaenicipitidae and Scopidae. These findings are consistent with recent molecular studies^16^^,^ ^71^ ^,^ ^72^ ^,^ ^73^ ^,^ 69 ^,^ 67 ^,^ 70 ^,^ 71 ^,^ 72 ^,^ 73 in which it is proposed that the “Pelecanidae” group retains the name Pelecaniformes while the second group be given the name “Phalacrocoraciiformes”^74^. Some analyses also place Threskiornithidae with the Pelecaniformes which would result in only the Ciconiidae remaining in the Ciconiiformes. The placement here of Threskiornithidae + Ciconiidae may simply reflect the recent state of flux of these taxa.

The sister group relationship of Sphenisciformes + Procellariiformes has support from both morphology^68^ and molecular data^16^. There is limited evidence for the placing of Gaviiformes with these taxa^75^ but the relative positions of all three orders within the “waterbird” assemblage is far from resolved. There are a number of well-known fossil penguins (e.g., *Delphinornis*, *Marambiornis*, *Perudyptes*) which are all placed basally within the Sphenisciformes in the supertree. The Procellariiformes consist of well established monophyletic families.

The Phoenicopteriformes + Podicipediformes clade found by the supertree, termed “Mirandornithes” by Sangster^76^, is well supported by a large number of molecular studies^16^^,^ 67 ^,^ 69 ^,^ 72 ^,^ 77 ^,^ 78.

Gruiformes/Otididae/Charadriiformes

The positioning of the Gruiformes + Otidae and the Charadriiformes (including the Turnicidae) as sister groups to the “waterbird” assemblage is congruent with recent molecular hypotheses^16^^,^ 67 ^,^ 73. The Turnicidae were traditionally placed in the Gruiformes^9^^,^ 79 but are now understand to be part of the Charadriiformes^63^. It is less certain that the Cariamidae are genuinely part of the Gruiformes + Otididae clade. The Cariamidae were also part of the traditional “Gruiformes” but may actually be more closely related to the falconiform birds^61^. The core Gruiformes found here are composed of the Psophidae, Gruidae, Heliornithidae, Aramidae and Rallidae. Within the Charadriiformes the most basal lineages include the plovers and allies; Chionidiae, Burhinidae, Pluvianidae, Recurvirostridae, Ibidorhynchidae, Haematopidae and Charadriidae. The supertree divides the remaining Charadriiformes into monophyletic gull and sandpiper lineages.

Columbiformes/Mesitornithidae/Eurypygidae

Eurypygidae + Rhynchochetidae is well supported by morphological and molecular data^16^^,^ 67 ^,^ 68 ^,^ 80 ^,^ 81 ^,^ 82. The Columbidae + Pteroclididae is less certain but relatively well established^83^^,^ 84. The position of these taxa with the Mesitornithidae as part of the “Metaves” is suggested by recent molecular studies^16^^,^ 67. The supertree however does not support the “Metaves” clade and it has been suggested that the “Metaves” does not constitute a monophyletic group as discussed in Mayr^61^. The results found here are more congruent with Chojnowski *et al*.'s^80^ findings of an affinity between Columbiformes and the core Gruiformes + “waterbird” assemblage. The supertree places the extinct Raphidae (dodo and solitaire) within the Columbiformes.

Musophagiformes/Opisthocomus

The relationships of the hoatzin are controversial and poorly understood. *Opisthocomus* has previously been placed with the Cuculiformes (cuckoos, coucals and anis)^85^^,^ 86 ^,^ 87, the Gruiformes (crakes and rails) and Musophagiformes^88^^,^ 89. The supertree supports the *Opisthocomus* + Musophagiformes relationship. Other putative close relatives include the Columbiformes^63^ but the supertree does not recover this relationship.

Caprimulgiformes/Apodiformes

Mayr^90^ coined the term “Strisores” for the clade containing Caprimulgiformes and Apodiformes that has received a great deal of support from molecular data^16^^,^ 67 ^,^ 71 ^,^ 90. The supertree supports the monophyly of this proposed clade and places it as sister to the “landbird” assemblage as in Pratt *et al.* 2009^91^; rather than as part of the “Metaves” as proposed elsewhere^16^^,^ 67 ^,^ 70 or as a polyphyletic group^73^. The sister group relationship of the “caprimulgiform” taxon Aegothelidae and the Apodiformes, resulting in a paraphlyletic “Caprimulgiformes” is well-supported by molecular and morphological data^16^^,^ 67 ^,^ 69 ^,^ 90 ^,^ 91 ^,^ 92 ^,^ 93 ^,^ 94. The Apodiformes contain a monophyletic Apodidae and Trochilidae, as in Sibley and Ahlquist’s^9^ “Trochiliformes” for hummingbird taxa. The association between the Apodiformes and “Trochiliformes” has long been recognised^63^^,^ 93 ^,^ 95 ^,^ 96 and is not contradicted by any of the source trees.

Falconiformes/Accipitriformes/Strigiformes

The Falconiformes and Accipitriformes represent a single lineage in the supertree. The Falconiformes and Strigiformes are united as in analyses based on osteology^68^^,^ 97. They are not however placed within the “landbird” assemblage as in recent large molecular studies^16^^,^ 67. New World vultures (Cathartidae) are not placed with the Ciconiiformes as in some early works but neither are they placed with the Old World Vultures (Accipitriformes)^16^^,^ 67 supporting the proposal that they might require an order level designation (“Cathartiformes”)^67^.

Coraciiformes/Alcediniformes/Piciformes/Psittaciformes

Recent large molecular analyses have proposed a “landbird” clade, the supertree recovers part of this clade but not in its entirety. The supertree does support a monophyletic clade containing the Coraciiformes, Alcediniformes and Piciformes which is well supported by molecular data^16^^,^ 67. The affinities of the Leptosomatidae are not well-understood^61^, the supertree places them within this “landbird” assemblage with the Coraciiformes. The Trogoniformes are a taxon for which higher level relationships are poorly understood, in the supertree they are placed as sister to the Coraciiformes + Alcediniformes + Piciformes clade with the Coliiformes and Cuculiformes also placed in this clade. The former is supported by molecular data^68^^,^ 98, however the latter is not well-supported.

The Piciformes are split into two distinct clades, one supporting the division into the monophyletic families Ramphastidae, Capitonidae, Megalaimidae (previously included within Capitonidae), Lybiidae and Semnornithidae^99^^,^ 100 ^,^ 101 ^,^ 102 and the second containing the monophyletic Picidae (woodpeckers) and the Indicatoridae (honeyguides) as in Simpson and Cracraft^101^, Swiersczewski and Raikow^102^ and Lanyon and Zink^100^.

The coraciiform clade contains the Brachypteraciidae, Coraciidae, Meropidae, Alcedinidae, Todidae and Momotidae. The Bucerotiformes, Bucorvidae, Bucerotidae and Phoeniculidae, are placed in a clade sister to the Piciformes. The Hoopoe, *Upupa epops*, is also placed within the Bucerotiformes in contrast to Sibley and Ahlquist’s^9^ suggestion of elevating it to a new order “Upupiformes”.

The Psittaciformes are traditionally considered to have no close living relatives^9^ but the supertree is consistent with more recent analyses that place them as the sister taxon to the Passeriformes^16^^,^ 67 ^,^ 69 ^,^ 89.

Passeriformes

The Passeriformes contain the majority of extant bird species and have undergone extensive reorganisation within the last decade. The supertree supports the division into three suborders: New Zealand Wrens (Acanthisitti) + all other passeriformes (Tyranni + Passeri). Monophyly of the Old and New World suboscines is well-documented^103^^,^ 104 and as expected the supertree splits the Tyranni (suboscines) into Old World (Eurylaimides) and New World (Tyrannides) groups, all of which contain well-established monophyletic families, the one exception being the Eurylaimidae which is now understood to be polyphletic^105^. In the supertree *Smithornis* and *Calpytomena* fall outwith the main Eurylaimidae clade. The neotropical *Sapayoa* *aenigma* was traditionally placed in the New World suboscines but has more recently been placed in the Old World suboscines in varying positions; the supertree places it at the base of the main Eurylaimidae clade (containing *Eurylaimus*)^106^^,^ 107 ^,^ 108. The New World suboscines are further split into two monophyletic superfamilies; the “bronchophone” suboscines and the Furnarioidea. The Oligocene fossils *Zygodactylus* and *Primozygodactylus danielsi* are placed at the base of the Passeriformes.

Sibley and Ahlquist^9^ split the Passeri into the Corvida and the Passerida but while the Passerida is retained it is now known that the “Corvida” do not comprise a monophyletic group^69^^,^ 109 ^,^ 110 ^,^ 111. Basal within the Passeri are the Menuridae and Atrichornithidae, sometimes designated as the superfamily Menuroidea^9^^,^ 112. The supertree also supports the superfamily status of the previously incertae sedis Ptilonorhynchoidea (Climacteridae + Ptilonorhynchidae)^9^^,^ 112 ^,^ 113, and supports a relationship between Orthonychidae + Pomatostomidae. The Meliphagoidea contains a monophyletic Maluridae, Pardalotidae, Acanthizidae and Meliphagidae.

The large well-supported^110^ superfamily Corvoidea includes the corvid birds that have radiated out from the Australo-Papuan region and diversified worldwide. As found in the previously published oscine supertree^34^ *Melanocharis* and *Paramythia* berrypeckers, and *Toxorhamphus* longbills appear to belong to Corvoidea rather than to Passeroidea as suggested by Sibley and Ahlquist^9^ and Monroe and Sibley^1^. Other lineages placed within this clade include well-established members of the core Corvoidea. These include the Campephagidae, Paradisaeidae, Monarchidae, Oriolidae, Dicuridae, Laniidae and Corvidae. The Picarthatidae + Chaetopidae + Eupetidae clade (possible superfamily) and Petroicidae are at the base of the large infraorder Passerida. This placing of the Petroicidae reflects recent views on their position within the oscine birds^110^^,^ 114.

The supertree supports the identification of a number of recently proposed superfamilies within the monophyletic Passerida clade in addition to Sibley and Ahlquist's^9^ original three: Sylvioidea, Muscicapoidea and Passeroidea. At the base of the Passerida are the Sylvioidea and the possible superfamily Paroidea. The Hyliotidae have recently been split from Sylviidae^115^ and are placed as sister to the Sylvioidea in the supertree. The Sylvioidea families have undergone a great deal of change in recent years, the supertree supports many of the newly suggested families and new delimitation of traditional families, for example the splitting of the “Timaliidae” into a core timaliid clade and a number of newly recognised lineages such as the Pellorneidae and Leiothrichidae^116^ and the splitting of the “Sylviidae” to recognise new families such as the Locustellidae and the Cisticoliidae^117^^,^ 118. Well-supported members of the Sylvioidea include Alaudidae, Hirundinidae, and Pycnonotidae^109^^,^ 117 ^,^ 119 ^,^ 120, while the supertree supports the inclusion of the Zosteropidae within the Timaliidae^34^.

The Muscicapidoidea and Certhioidea form a clade with the proposed Bombycilloidea and Reguloidea superfamilies. Muscicapoidea intra-relationships are well-supported by a number of analyses^9^^,^ 109 ^,^ 115 ^,^ 118 ^,^ 119 ^,^ 121 and the supertree finds the traditional families Mimidae, Cinclidae, Sturnidae, Turdidae and Muscicapidae along with the Buphagidae and Rhabbdornithidae also being placed as distinct families.

The Passeroidea is the largest passeriform superfamily. Along with finches, sparrows, weavers etc. it contains the nine-primaried oscines - songbirds with nine easily identifiable primary feathers on each wing. The nine-primaried oscines are a large radiation that contains approximately 10% of all extant species of birds^122^ and form a strongly supported monophyletic clade^109^^,^ 110 ^,^ 122. The supertree does not place the Peucedramidae within the nine-primaried oscines but with the Prunellidae. All the families are resolved as monophyletic with the exception of the Thraupidae/Cardinalidae clade which has undergone extensive reorganisation in recent years^123^. The supertree was unable to resolve the position of the Icteridae, the varying position of the Icteridae in the supertree as sister to either the Parulidae or the Emberizidae both have support from recent analyses^109^^,^ 119 ^,^ 124. In the simplified family level tree we have collapsed these three families to a trichotomy to reflect this uncertainty, which seems likely to be a reflection of its varying position in source trees rather than a true biological relationship. The supertree supports the separation of the estrildid finches and the true sparrows into two families the Estrildidae and Passeridae as in Christidis and Boles^74^. The Dicaeoidea (Nectariniidae + Dicaeidae) and Promeropidae are at the base of the Passeroidea. These may represent independent superfamilies or may be included as part of the Passeroidea.

## Conclusions

The supertree is the first published species-level supertree of birds. It is also the first comprehensive phylogeny of birds to include fossils; both recently extinct and Mesozoic taxa, which are of vital importance for analyses requiring an understanding of the deep evolutionary history of birds. It is not intended to be the final word in avian systematics nor is it intended to be used as a basis for re-evaluating avian taxonomy. It does, however, provide a platform upon which further research can be based and will hopefully provide a useful resource for researchers studying avian macroevolution, conservation, biodiversity, comparative biology and character evolution. An earlier version of the supertree^24^ has already been used in a large number and variety of evolutionary studies^125^^,^ 126 ^,^ 127 ^,^ 128 ^,^ 129 ^,^ 130 ^,^ 131 ^,^ 132 ^,^ 133 ^,^ 134 and it is anticipated that this updated tree will provide a basis for further research of this nature and may be of particular use to macroevolutionary studies due to the inclusion of fossil taxa. We acknowledge that many additional papers have been published since our data collection ceased - avian systematics is a rapidly moving field. This tree does however represent a significant update compared to Davis^24^ and we anticipate that a further update will be published in the future; for now this tree is still the only large avian phylogeny available with a broad taxonomic coverage containing both fossil and extant taxa. This work highlights areas in which systematic knowledge is poor or inconsistent, suggesting a possible focus for future phylogenetic studies. We also identify the need for leaf-based measures of support to aid identification of rogue taxa in supertree analyses. The supertree represents a first attempt at a species-level avian supertree and will no doubt be improved upon as further data and better algorithms become available.

## Availability of supporting data

All supplementary data are available at figshare: http://dx.doi.org/10.6084/m9.figshare.976113

## Competing Interest Statement

The authors declare that no competing interests exist.
